# Bortezomib induced peripheral neuropathy and single nucleotide polymorphisms in *PKNOX1*

**DOI:** 10.1186/s40364-023-00490-9

**Published:** 2023-05-16

**Authors:** Xiang Zhou, Seungbin Han, Nadine Cebulla, Larissa Haertle, Maximilian J. Steinhardt, Daniel Schirmer, Eva Runau, Leon Flamm, Calvin Terhorst, Laura Jähnel, Cornelia Vogt, Silvia Nerreter, Eva Teufel, Emilia Stanojkovska, Julia Mersi, Umair Munawar, Magnus Schindehütte, Robert Blum, Ann-Kristin Reinhold, Oliver Scherf-Clavel, Heike L. Rittner, Mirko Pham, Leo Rasche, Hermann Einsele, Claudia Sommer, K. Martin Kortüm

**Affiliations:** 1 Department of Internal Medicine II, University Hospital of Würzburg, Würzburg, Germany; 2 Department of Neurology, University Hospital of Würzburg, Würzburg, Germany; 3 Department of Hematology, Spanish National Cancer Research Center (CNIO), Hospital Universitario 12 de Octubre, Complutense University Madrid, Madrid, Spain; 4 Department of Neuroradiology, University Hospital of Würzburg, Würzburg, Germany; 5 Center for Interdisciplinary Medicine, Department of Anesthesiology, Intensive Care, Emergency and Pain Medicine, University Hospital of Würzburg, Würzburg, Germany; 6 Department of Clinical Pharmacy, University of Würzburg, Würzburg, Germany

**Keywords:** PKNOX1, Multiple myeloma, Bortezomib, Peripheral neuropathy

## Abstract

We analyzed single nucleotide polymorphisms (SNPs) in *PKNOX1* (rs2839629) and in the intergenic region between *PKNOX1* and *CBS* (rs915854) by Sanger sequencing in 88 patients with multiple myeloma treated with bortezomib. All patients (n = 13) harboring a homozygous mutation in *PKNOX1* (rs2839629) also had a homozygous mutated rs915854 genotype. Homozygous mutated genotypes of rs2839629 and rs915854 were significantly enriched in patients with painful peripheral neuropathy (PNP) (*P* < 0.0001), and homozygous mutated rs2839629 genotype was significantly enriched in patients with pain compared to patients with no pain (*P* = 0.04). In summary, both SNPs rs2839629 and/or rs915854 may be potential biomarkers predicting an increased risk to develop painful PNP under bortezomib.

**Supplementary Information:**

The online version contains supplementary material available at 10.1186/s40364-023-00490-9.

## To the editor

The treatment of multiple myeloma (MM) is evolving rapidly. Although novel immunotherapies, e.g. antibody drug conjugate, bispecific antibody, and chimeric antigen receptor modified T-cell therapy are being incorporated into the standard of care, bortezomib (BTZ), the first-in-class proteasome inhibitor (PI), still presents the most widely used anti-MM agent especially in newly diagnosed (ND) MM [1]. Peripheral neuropathy is one of the most common non-hematologic side effects in patients treated with BTZ [2]. However, the underlying mechanism of BTZ induced peripheral neuropathy (BIPN) is not fully understood. A previous study suggested that single nucleotide polymorphisms (SNP) in PBX/Knotted 1 Homeobox 1 *(PKNOX1*) gene correlate with an increased risk to develop BIPN [3]. Therefore, we performed the current study to further elucidate the role of *PKNOX1* genotypes in the development of BIPN.

In this prospective study, we included 88 MM patients, in whom we analyzed SNPs in *PKNOX1* by Sanger sequencing. Patients’ demographics and MM-related data were collected. Additionally, we performed a neurological assessment to determine the severity of BIPN, and the neurological data were published elsewhere [4]. All procedures were in accordance with the Declaration of Helsinki as revised in 2013, and this study was approved by the ethics committee of Medical Faculty of the University of Würzburg, Germany. More details of the methods are available in the **supplementary information**.

In our cohort, the median age at BTZ start was 62 (range 30–79) years, and the majority of the patients was male (n = 64, 73%). Ten (11%) patients had just started BTZ treatment at the time point of study inclusion. Thirty-seven (42%) patients were under ongoing BTZ treatment, and in 41 (47%) patients, BTZ had been given but was already stopped prior to the inclusion in the current study. In addition, 27 (31%) patients had been treated with thalidomide. Preexisting idiopathic sensory-motor polyneuropathy and diabetic neuropathy were documented in 3 (3%) and 1 (1%) patients, respectively. One (1%) patient showed sensory-motor deficits after intracranial hemorrhage before the diagnosis of MM. In total, 73 (83%) patients presented BIPN, with 34 (39%) patients suffering from painful BIPN. Severe BIPN ≥ grade 3 was found in only 3 (3%) patients. The patients’ characteristics are summarized in Table 1.

**Table 1:** Patients’ characteristics

Parameter	
**Patients, n**	88
**Gender, n (%)** Male Female	64 (73) 24 (27)
**Age at diagnosis, median, years (range)** **Age at bortezomib start, median, years (range)**	61 (30–79) 62 (30–79)
**Subtype, n (%)** IgG Non-IgG LC	46 (52) 18 (21) 24 (27)
**Cytogenetics, n (%)** High-risk^#^ Standard-risk	18 (20) 70 (80)
**Patient groups, n (%)** Bortezomib naïve under ongoing bortezomib treatment s.p. bortezomib treatment^§^	10 (11) 37 (42) 41 (47)
**Prior treatment, n (%)** **Immunomodulatory drugs** Lenalidomide Pomalidomide Thalidomide **Proteasome inhibitors other than bortezomib** Carfilzomib Ixazomib **Monoclonal antibodies** Daratumumab Elotuzumab	48 (55) 26 (30) 27 (31) 22 (25) 5 (6) 65 (74) 9 (10)
**Pre-existing peripheral neuropathy, n (%)** Idiopathic sensory-motor polyneuropathy Diabetic neuropathy Sensory-motor deficits after intracranial hemorrhage	3 (3) 1 (1) 1 (1)
**Prevalence of BIPN, n (%)** No BIPN BIPN without pain Painful BIPN	15 (17) 39 (44) 34 (39)
**BIPN grade, n (%)** No BIPN Grade 1 Grade 2 Grade 3	15 (17) 44 (50) 26 (30) 3 (3)

BIPN - bortezomib induced peripheral neuropathy; LC - light chain; ^#^ defined as presence of at least one of the following: t(4;14), t(14;16), t(14;20), del(17p). ^§^ Bortezomib had been given but was already stopped prior to the inclusion in the current study

**Fig. 1:**
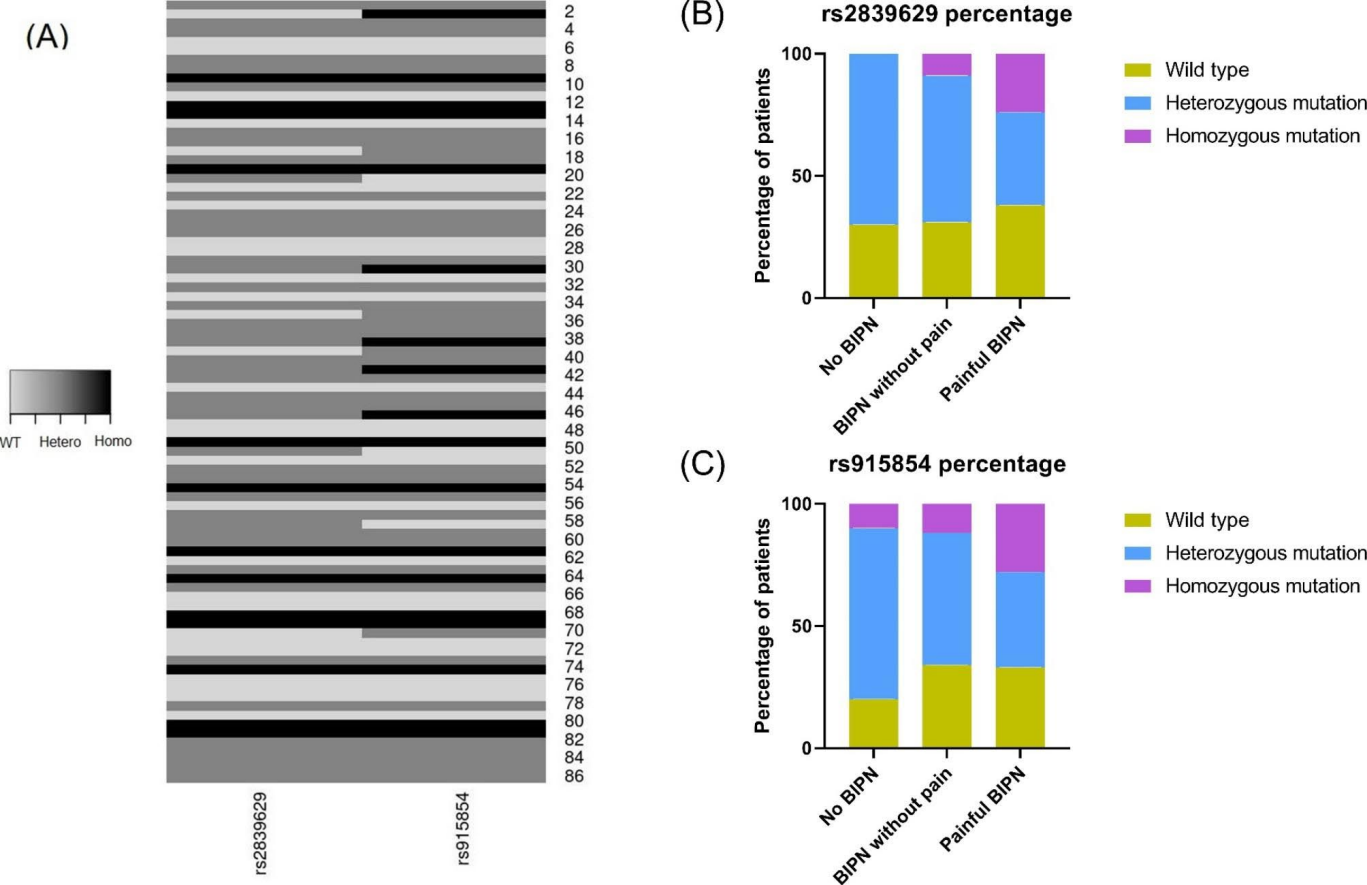
(A) demonstrates the frequencies of SNPs rs2839629 and rs915854. Wilde type: light grey; heterozygous mutation: dark grey; homozygous mutation: black. For rs2839629: wild type = G, heterozygous = A/G, homozygous = A; for rs915854: wild type = C, heterozygous = C/T, homozygous = T. (B-C) display the frequencies of SNPs rs2839629 (data evaluable in 77 patients; no BIPN: n = 10; BIPN without pain: n = 35; painful BIPN: n = 32) and rs915854 (data evaluable in 78 patients; no BIPN: n = 10; BIPN without pain: n = 35; painful BIPN: n = 33) in different patient subgroups. Homozygous mutated rs2839629 and rs915854 genotypes were significantly enriched in painful BIPN (for both: Chi-square test *P* < 0.0001). BIPN - bortezomib induced peripheral neuropathy; Hetero - heterozygous; Homo - homozygous; WT - wild type

We investigated the association between BIPN and SNPs in the *PKNOX1* gene (rs2839629) and in the intergenic region between *PKNOX1* and cystathionine-ß-synthetase (*CBS*) (rs915854). Genotypes of SNPs rs2839629 and rs915854 were evaluable in 86 and 88 patients, respectively. Among the 86 patients with genotypes of both rs2839629 and rs915854, we noticed that all patients (n = 13) harboring a homozygous mutation in the *PKNOX1* gene (rs2839629) also showed a homozygous mutated rs915854 genotype. Moreover, the vast majority of the 44 patients with heterozygous *PKNOX1* gene mutation (rs2839629) displayed mutated rs915854 genotype (homozygous: n = 4; heterozygous: n = 36; wild type: n = 4), suggesting a high rate of co-mutated rs2839629 and rs915854 (Fig. 1A, Table S1). Notably, the minor allele frequencies of SNPs rs2839629 and rs915854 in MM patients were similar to that in the general population in Europe (Table S2) [3].

We then divided the 78 patients who had been treated with BTZ into three groups: no BIPN, BIPN without pain, and painful BIPN. The frequencies of SNPs rs2839629 and rs915854 were compared among these three patient subgroups. Notably, homozygous mutated genotypes of rs2839629 and rs915854 were significantly enriched in patients with painful BIPN (for both SNPs: Chi-square test *P* < 0.0001) (Fig. 1B-C). Moreover, homozygous mutated rs2839629 genotype was significantly more common in patients with pain when compared to patients with no pain (Fisher exact test *P* = 0.04) (Figure S1B). Similarly, we observed a tendency that homozygous mutated rs915854 genotype was enriched in painful BIPN (Fisher exact test *P* = 0.08) (Figure S1D). Moreover, the frequency of homozygous *PKNOX1* mutation (rs2839629) was significantly higher in the patients, who required BTZ dose reduction, compared with that in the remaining patients (Fisher exact test *P* = 0.03) (Figure S1E). Interestingly, in our cohort, thalidomide treatment did not significantly impact the development of peripheral neuropathy (Fisher exact test *P* = 0.44) and pain (Fisher exact test *P* = 0.79) (Figure S1G-H). In addition, patients with BIPN had received higher BTZ cumulative dose compared with those without BIPN (Mann-Whitney U-test *P* = 0.03), underlining that cumulative BTZ dosing may influence the development of BIPN (Figure S2).

A previous genome-wide association study suggested a significant association between BIPN and *PKNOX1* genotype [3]. *PKNOX1* is a transcription modulator of monocyte chemoattractant protein 1 (MCP-1) gene, and increased *PKNOX1* expression was seen in neuropathic pain [3, 5]. SNPs rs2839629 and rs915854 were significantly associated with *PKNOX1* expression in nerve tissue, explaining the relationship between rs2839629 or rs915854 genotype and painful BIPN In our study, we found that homozygous mutated rs2839629 or rs915854 genotypes were significantly enriched in patients with painful BIPN. Additionally, we noticed a high rate of simultaneous mutation of both rs2839629 and rs915854 in our cohort. Therefore, screening of SNPs rs2839629 and/or rs915854 prior to BTZ start may provide useful information for the prediction of BIPN. Close monitoring should be performed especially in patients with homozygous genotype, which may require BTZ dose reduction during the treatment.

At present, D-VTd (daratumumab, BTZ, thalidomide, and dexamethasone) represents the most commonly used induction chemotherapy for transplant eligible NDMM in Europe [6]. However, in this regimen, both BTZ and thalidomide are neurotoxic agents and, indeed, peripheral sensory-motor neuropathy was significantly higher in the CASSIOPEIA trial evaluating D-VTd compared to the CASTOR trial in which the D-Vd combination (daratumumab, BTZ, and dexamethasone) was tested (all grade: 60.9% versus 48.1%, Fisher exact test *P* < 0.0001; ≥ grade 3: 8.6% versus 5.6%, Fisher exact test *P* = 0.04) [6, 7]. Of interest, in our study, thalidomide treatment did not increase the prevalence of sensory-motor neuropathy. This may be explained by the limited number of thalidomide treated cases in our cohort, but also by the known differences in pathogenesis of BTZ versus thalidomide induced peripheral neuropathy [8].

BIPN is a frequent and clinically relevant problem in the treatment of MM, requiring dose reduction and leading to a poorer quality of life. Patients with homozygous mutated genotype in *PKNOX1* (rs2839629) and/or in the intergenic region between *PKNOX1* and *CBS* (rs915854) have an increased risk to develop BIPN, and that *PKNOX1* plays an important role in the multi-factorial process of BIPN development. Other potential pathomechanisms are currently explored in our ongoing clinical research unit (KFO5001), such as epigenetic modifications and neurofilament light chain levels [4, 9, 10] to better understand BIPN development and resolution.

## Electronic supplementary material

Below is the link to the electronic supplementary material.

Supplementary Material 1

## Data Availability

All data, if not given in this article, is available on request.
