# Supplementary material for: Bortezomib induced peripheral neuropathy and single nucleotide polymorphisms in PKNOX1
Source: Biomark Res. 2023 May 16;11:52. doi: 10.1186/s40364-023-00490-9 (PMC10189922; doi:10.1186/s40364-023-00490-9)
Supplement: Supplementary file 1 — Supplementary Material 1 [file 40364_2023_490_MOESM1_ESM.docx]

**Supplementary information:**

**Bortezomib induced peripheral neuropathy and single nucleotide polymorphisms in *PKNOX1***

Xiang Zhou^1$^, Seungbin Han^1$^, Nadine Cebulla^2^, Larissa Haertle^1,3^, Maximilian J. Steinhardt^1^, Daniel Schirmer^2^, Eva Runau^2^, Leon Flamm^2^, Calvin Terhorst^2^, Laura Jähnel^2^, Cornelia Vogt^1^, Silvia Nerreter^1^, Eva Teufel^1^, Emilia Stanojkovska^1^, Julia Mersi^1^, Umair Munawar^1^, Magnus Schindehütte^4^, Robert Blum^2^, Ann-Kristin Reinhold^5^, Oliver Scherf-Clavel^6^, Heike L. Rittner^5^, Mirko Pham^4^, Leo Rasche^1^, Hermann Einsele^1^, Claudia Sommer^2^, K. Martin Kortüm^1^

^1^ Department of Internal Medicine II, University Hospital of Würzburg, Würzburg, Germany

^2^ Department of Neurology, University Hospital of Würzburg, Würzburg, Germany

^3^ Department of Hematology, Hospital Universitario 12 de Octubre, Spanish National Cancer Research Center (CNIO), Complutense University Madrid, Madrid, Spain

^4^ Department of Neuroradiology, University Hospital of Würzburg, Würzburg, Germany

^5^ Center for Interdisciplinary Medicine, Department of Anesthesiology, Intensive Care, Emergency and Pain Medicine, University Hospital of Würzburg, Würzburg, Germany

^6^ Department of Clinical Pharmacy, University of Würzburg

^$^ contributed equally

**Corresponding author:**

K. Martin Kortüm, MD

Department of Internal Medicine II

University Hospital of Würzburg

Würzburg, Germany

E-mail: kortuem_m@ukw.de

**Supplementary methods**

**Patients and treatment**

We collected peripheral blood samples from 88 multiple myeloma (MM) patients who had either just started bortezomib (BTZ) treatment, were under ongoing BTZ treatment, or had stopped BTZ at an earlier time point of their disease history (s.p. BTZ treatment). BTZ was given subcutaneously (SC) at a dosage of 1.3 mg/m^2^ on days 1, 4, 8, and 11 in each cycle, and was repeated on day 28. The grade of BTZ induced peripheral neuropathy (BIPN) was classified according to the Common Terminology Criteria for Adverse Events (CTCAE) Version 5.0.

**Sample processing and genotyping**

Patients’ peripheral blood samples were collected with EDTA tubes. Peripheral blood mononuclear cells (PBMCs) were isolated via Ficoll-Hypaque density gradient centrifugation followed by DNA extraction using Allprep DNA/RNA Mini kit (Qiagen), according to the manufacturer’s instructions. To PCR amplify regions containing the Single Nucleotide Polymorphisms (SNPs) rs2839629 and rs915854, two primer pairs F1 (5′-TAGAGAAAGGGTGCTTATGGACAT-3′) and R1 (5′-AACGAAATCTTAGAGGGGGTCA-3′), F2 (5′-CGAGCAGATCCTGCACAAAG-3′) and R2 (5′-TGGTACATTTTACCACAGTGCG-3′) were used, respectively. The genotyping of the SNPs rs2839629 and rs915854 was assessed with commercially available Sanger sequencing from LGC Genomics, applying both PCR primer pairs. The .Ab1 files were then aligned using the SnapGene software 5.0.8 (GSL Biotech LLC, San Diego, CA, USA) and compared with the reference sequence, extracted from the ENSEMBl genome browser (GRCh38.p13).

**Statistical analysis**

We applied Fisher´s exact test to investigate the association between the SNPs rs2839629 or rs915854 and the development of BIPN or pain. The Chi-square test was used to compare the frequencies of the SNPs rs2839629 and rs915854 in different subgroups. We used Mann-Whitney U-test to compare the cumulative BTZ doses in subgroups. All statistical analyses were performed using GraphPad Prism software 9.4.1 (GraphPad Software, Inc, San Diego, CA, USA).

**Supplementary Tables**

**Table S1: Genotypes of SNPs rs2839629 and rs915854**

| **No** | **rs2839629** | **rs915854** |
| --- | --- | --- |
| 1 | A/G | C/T |
| 2 | G | T |
| 3 | A/G | C/T |
| 4 | A/G | C/T |
| 5 | G | C |
| 6 | G | C |
| 7 | A/G | C/T |
| 8 | A/G | C/T |
| 9 | A | T |
| 10 | A/G | C/T |
| 11 | G | C |
| 12 | A | T |
| 13 | A | T |
| 14 | G | C |
| 15 | A/G | C/T |
| 16 | A/G | C/T |
| 17 | G | C/T |
| 18 | A/G | C/T |
| 19 | A | T |
| 20 | A/G | C |
| 21 | G | C |
| 22 | A/G | C/T |
| 23 | G | C |
| 24 | A/G | C/T |
| 25 | A/G | C/T |
| 26 | A/G | C/T |
| 27 | G | C |
| 28 | G | C |
| 29 | A/G | C/T |
| 30 | A/G | T |
| 31 | G | C |
| 32 | A/G | C/T |
| 33 | G | C |
| 34 | A/G | C/T |
| 35 | G | C/T |
| 36 | A/G | C/T |
| 37 | A/G | C/T |
| 38 | A/G | T |
| 39 | G | C/T |
| 40 | A/G | C/T |
| 41 | A/G | T |
| 42 | A/G | C/T |
| 43 | G | C |
| 44 | A/G | C/T |
| 45 | A/G | C/T |
| 46 | A/G | T |
| 47 | G | C |
| 48 | G | C |
| 49 | A | T |
| 50 | A/G | C |
| 51 | G | C |
| 52 | A/G | C/T |
| 53 | A/G | C/T |
| 54 | A | T |
| 55 | A/G | C/T |
| 56 | G | C |
| 57 | A/G | C/T |
| 58 | A/G | C |
| 59 | A/G | C/T |
| 60 | A/G | C/T |
| 61 | A | T |
| 62 | G | C |
| 63 | A/G | C/T |
| 64 | A | T |
| 65 | A/G | C/T |
| 66 | G | C |
| 67 | G | C |
| 68 | A | T |
| 69 | A | T |
| 70 | G | C/T |
| 71 | G | C |
| 72 | G | C |
| 73 | A/G | C/T |
| 74 | N/A | C/T |
| 75 | A | T |
| 76 | G | C |
| 77 | G | C |
| 78 | G | C |
| 79 | A/G | C/T |
| 80 | N/A | C/T |
| 81 | G | C |
| 82 | A | T |
| 83 | A | T |
| 84 | A/G | C/T |
| 85 | A/G | C/T |
| 86 | A/G | C/T |
| 87 | A/G | C/T |
| 88 | A/G | C |

N/A - not available; for rs2839629: wild type=G, heterozygous=A/G, homozygous=A; for rs915854: wild type=C, heterozygous=C/T, homozygous=T.

**Table S2: Minor allele frequency (MAF) of SNPs rs2839629 and rs915854 (Magrangeas et al. 2016)**

| **MAF of rs2839629 in MM patients in our cohort** | | **MAF of rs2839629 in general population (European subgroup)** | | **MAF of rs2839629 in general population (global)** | |
| --- | --- | --- | --- | --- | --- |
| G | 0.597 | G | 0.558 | G | 0.551 |
| A | 0.403 | A | 0.442 | A | 0.449 |
| **MAF of rs915854 in MM patients in our cohort** | | **MAF of rs915854 in general population (European subgroup)** | | **MAF of rs915854 in general population (global)** | |
| C | 0.444 | C | 0.451 | C | 0.431 |
| T | 0.556 | T | 0.549 | T | 0.569 |

**Supplementary figures**

**Figure S1:** (A) SNP rs2839629 in patients with versus without BIPN, *P*=0.34; (B) SNP rs2839629 in patients with versus without pain, *P*=0.04*; (C) SNP rs915854 in patients with versus without BIPN, *P*=0.68; (D) SNP rs915854 in patients with versus without pain, *P*=0.08; (E) SNP rs2839629 in patients with versus without BTZ dose reduction, *P*=0.03*; (F) SNP rs915854 in patients with versus without BTZ dose reduction, *P*=0.34; (G-H) Role of thalidomide in the development of BIPN (*P*=0.44) and pain (*P*=0.79) (BTZ-naïve patients were excluded). BIPN - bortezomib induced peripheral neuropathy; BTZ - bortezomib; SNP - single nucleotide polymorphism; * Fisher’s exact test, a *P*-value <0.05 was considered to be statistically significant.


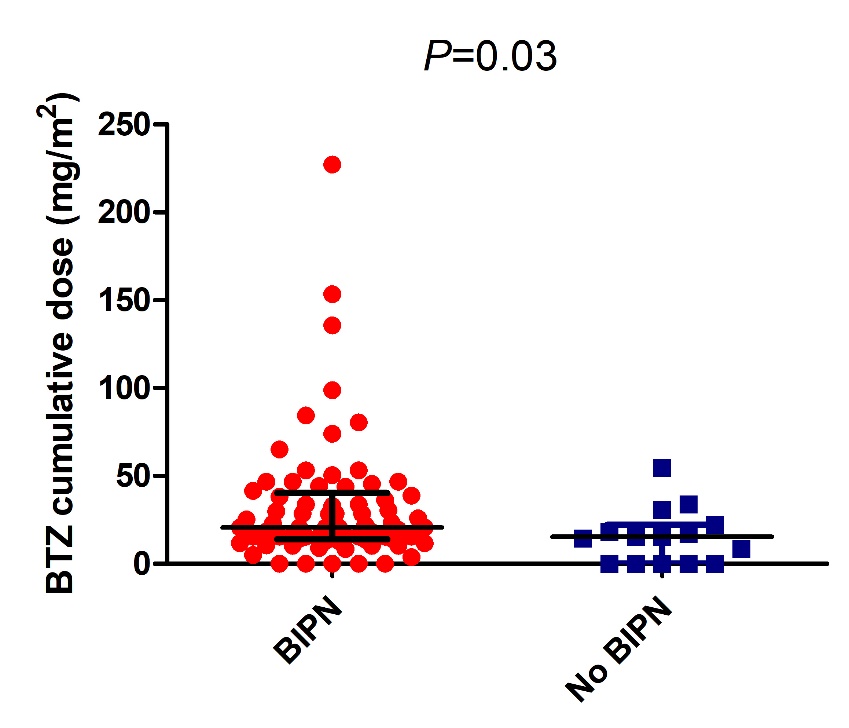


**Figure S2:** Cumulative BTZ dose was higher in patients with BIPN compared to those without BIPN (*P*=0.03). BTZ – bortezomib, BIPN – bortezomib induced peripheral neuropathy, bars indicated median and 95% CI.

**References**

1. Magrangeas, F., R. Kuiper, H. Avet-Loiseau, W. Gouraud, C. Guerin-Charbonnel, L. Ferrer, A. Aussem, H. Elghazel, J. Suhard, H. Sakissian, M. Attal, C. Munshi N, P. Sonneveld, C. Dumontet, P. Moreau, M. van Duin, L. Campion, and S. Minvielle. 2016. 'A Genome-Wide Association Study Identifies a Novel Locus for Bortezomib-Induced Peripheral Neuropathy in European Patients with Multiple Myeloma', *Clin Cancer Res*, 22: 4350-55.
